# A novel genetic variant of *Streptococcus pneumoniae* serotype 11A discovered in Fiji

**DOI:** 10.1016/j.cmi.2017.06.031

**Published:** 2018-04

**Authors:** S. Manna, B.D. Ortika, E.M. Dunne, K.E. Holt, M. Kama, F.M. Russell, J. Hinds, C. Satzke

**Affiliations:** 1) Pneumococcal Research, Murdoch Childrens Research Institute, Royal Children's Hospital, Parkville, Victoria, Australia; 2) Centre for Systems Genomics, The University of Melbourne, Parkville, Victoria, Australia; 3) Department of Paediatrics, The University of Melbourne, Parkville, Victoria, Australia; 4) Department of Biochemistry and Molecular Biology, Bio21 Molecular Science and Biotechnology Institute, The University of Melbourne, Parkville, Victoria, Australia; 5) Department of Microbiology and Immunology at the Peter Doherty Institute for Infection and Immunity, The University of Melbourne, Parkville, Victoria, Australia; 6) Centre for International Child Health, Murdoch Childrens Research Institute, Melbourne, Australia; 7) Ministry of Health and Medical Services, Suva, Fiji; 8) Institute for Infection and Immunity, St. George's, University of London, United Kingdom; 9) BUGS Bioscience, London Bioscience Innovation Centre, London, United Kingdom

**Keywords:** Atypical pneumococcal serotype, Genetic variant, Pneumococcal capsule, Pneumococcal serotyping, Pneumococcus, *Streptococcus pneumoniae*

## Abstract

**Objectives:**

As part of annual cross-sectional *Streptococcus pneumoniae* carriage surveys in Fiji (2012–2015), we detected pneumococci in over 100 nasopharyngeal swabs that serotyped as ‘11F-like’ by microarray. We examined the genetic basis of this divergence in the 11F-like capsular polysaccharide (*cps*) locus compared to the reference 11F *cps* sequence. The impact of this diversity on capsule phenotype, and serotype results using genetic and serologic methods were determined.

**Methods:**

Genomic DNA from representative 11F-like *S. pneumoniae* isolates obtained from the nasopharynx of Fijian children was extracted and subject to whole genome sequencing. Genetic and phylogenetic analyses were used to identify genetic changes in the *cps* locus. Capsular phenotypes were evaluated using the Quellung reaction and latex agglutination.

**Results:**

Compared to published 11F sequences, the *wcwC* and *wcrL* genes of the 11F-like *cps* locus are phylogenetically divergent, and the *gct* gene contains a single nucleotide insertion within a homopolymeric region. These changes within the DNA sequence of the 11F-like *cps* locus have modified the antigenic properties of the capsule, such that 11F-like isolates serotype as 11A by Quellung reaction and latex agglutination.

**Conclusions:**

This study demonstrates the ability of molecular serotyping by microarray to identify genetic variants of *S. pneumoniae* and highlights the potential for discrepant results between phenotypic and genotypic serotyping methods. We propose that 11F-like isolates are not a new serotype but rather are a novel genetic variant of serotype 11A. These findings have implications for invasive pneumococcal disease surveillance as well as studies investigating vaccine impact.

## Introduction

*Streptococcus pneumoniae* (the pneumococcus) is a leading cause of morbidity and mortality worldwide [1]. There are over 90 pneumococcal serotypes, classified by an immunologically distinct capsule encoded by the capsular polysaccharide (*cps*) biosynthesis locus of the genome. The capsule is a major virulence factor and the basis for the currently licensed vaccines targeted toward the pediatric and elderly communities.

Pneumococcal colonization of the nasopharynx, most common in children younger than five years old, is considered a precursor to disease [2]. From the nasopharynx, pneumococci can disseminate to other anatomic sites to cause diseases such as pneumonia, otitis media and meningitis [3], [4], [5]. As pneumococcal conjugate vaccines (PCVs) reduce carriage of vaccine serotypes, they provide indirect benefits to unvaccinated individuals because nasopharyngeal carriage also underpins host-to-host transmission [3], [4], [5], [6].

As a result of both their direct and indirect protective effects, PCVs play a crucial role in reducing the burden of pneumococcal disease [7]. However, PCV introduction provides the opportunity for serotypes not targeted by the vaccine (non-vaccine-type pneumococci) to occupy the ecologic niche that vaccine types leave behind. This is referred to as serotype replacement. As a result, non-vaccine-type pneumococci are increasingly reported in carriage and disease after PCV introduction [8]. Serotyping is therefore important for monitoring serotype replacement in carriage and disease surveillance. A number of pneumococcal serotyping methods exist, with the reference standard considered to be the Quellung reaction [9], a serologic approach that involves mixing pneumococci with various antisera that recognize serogroup/type-specific capsular antigens, and visualizing the reaction under the microscope [10]. However, this method is laborious and requires experienced microbiologists for interpretation.

Pneumococcal serotyping can be complicated by genetic variants that exist within a specific serotype, especially when molecular methods are used. For example, the identification of genetic *cps* locus variants within serotype 6B led to a new serotype (6E) being proposed. However, subsequent work confirmed the divergent 6E *cps* locus still encodes a 6B capsule polysaccharide [11]. In contrast, serotype 11E was originally considered a genetic variant of 11A until differences in the antigenic properties of 11E and 11A capsules were identified, confirming 11E as a new serotype. This change in phenotype is due to nonsense mutations within the *wcjE* gene [12]. More recently, nonsense mutations have been identified in the *wciG* gene of serotype 35B invasive isolates. This results in variants that can no longer be serotyped by Quellung reaction and have been proposed as a new serotype (35D) [13], [14]. Because some variations in *cps* sequence lead to changes in phenotype while others do not, it is important to have a detailed understanding of such variants and how they can affect serotyping results.

Recently our group conducted a comprehensive study that compared 20 pneumococcal serotyping methods focusing on serotype detection from clinical samples [15]. Microarray, a genomics-based molecular approach that determines serotype based on the *cps* gene content of DNA extracts, was the best-performing method. We applied the microarray to nasopharyngeal samples from children as part of a vaccine impact study in Fiji, where PCV10 was introduced into the national immunization schedule in 2012. Microarray identified 106 pneumococci as ‘11F-like,’ indicating the *cps* locus in these isolates was most similar to that of serotype 11F but with divergence detected in the *cps* locus. In this study, we aimed to determine firstly the genetic basis of the sequence divergence in the 11F-like *cps* locus of *S. pneumoniae* isolates from Fiji, and secondly whether these differences translate to a change in phenotype (capsular structure), influencing serotyping results.

## Methods

### Nasopharyngeal swab collection and screening for pneumococci

As part of annual cross-sectional pneumococcal carriage surveys in Fiji, nasopharyngeal swabs were collected from study participants according to World Health Organization guidelines [9]. Ethical approval for this study was obtained from the Fiji National Research ethics review committee and the University of Melbourne Human research ethics committee, with written informed consent obtained from all the participants or their caregivers. Participants were from four age groups: 5- to 8-week-old infants, 12- to 23-month-old children, 2- to 7-year-old children and adult caregivers. Swabs were placed in 1 mL skim milk, tryptone, glucose and glycerol media [16] and kept in a cool box until transport to the Fiji Centre for Communicable Disease Control, where they were aliquoted and stored at −80°C. Samples were then shipped to the Murdoch Childrens Research Institute on dry ice and stored at −80°C until use. DNA was extracted from 100 μL aliquots of the nasopharyngeal swabs and screened for the presence of pneumococcal DNA using quantitative real-time PCR (qPCR) targeting the *lytA* gene as previously described [17].

### Molecular serotyping by microarray

For samples in which *lytA* was detected, a previously unthawed aliquot of the original nasopharyngeal swab was plated on horse blood agar containing gentamicin (5 μg/mL, to select for pneumococci) and incubated overnight at 37°C with 5% CO_2_. Bacteria were collected from plates containing α-hemolytic growth using 1 mL phosphate-buffered saline. DNA was extracted from the bacterial suspensions using the QIAcube HT with the QIAamp 96 DNA QIAcube HT Kit (Qiagen). Molecular serotyping by microarray was performed as described previously [15]. Briefly, the Genomic DNA ULS Labeling Kit (Agilent Technologies) was used to label 200 ng of DNA with a fluorescent probe (either Cy3 or Cy5) by incubating the reactions at 85°C in a thermocycler with a heated lid for 30 minutes. After purification of labelled DNA, the samples were incubated overnight at 65°C rotating at 20 rpm with Senti-SPv1.5 microarray slides (BUGS Bioscience) to allow hybridization to occur. Microarray slides were subsequently washed, scanned and analysed using Agilent microarray scanner and feature extraction software. Data were analysed by Senti-NET software to determine serotype calls, which were calculated using Bayesian-based models [18].

### Bacterial isolates

*S. pneumoniae* 11F-like isolates used in this study (PMP1342 and PMP1343) were purified from randomly selected nasopharyngeal swabs found to contain an 11F-like serotype by microarray (Supplementary Table S1). α-hemolytic colonies were isolated on horse blood agar supplemented with gentamicin (5 μg/mL) and confirmed as *S. pneumoniae* using optochin sensitivity testing and whole genome sequencing.

### Whole genome sequencing

Genomic DNA was extracted from pneumococcal isolates as described above. DNA was sequenced in 2 × 250 bp paired end reads using the MiSeq platform. *De novo* assembly was performed using SPAdes [19] and annotated with RAST [20].

### Sequence analysis

Phylogenetic analyses were performed using the software package MEGA 6 [21]. The *cps* locus gene sequences from 11F-like isolates and other serogroup 11 strains (GenBank accession nos. GU074952, CR931654, CR931655, CR931656, GU074953, CR931657) were aligned using MUSCLE. Maximum likelihood trees were then generated from the alignment using the Tamura-Nei model. Statistical support for the branches was ascertained by bootstrapping (1000 replicates). For the identification of single residue substitutions, insertions or deletions, 11F-like *cps* sequences were aligned to known 11A and 11F sequences using Clustal Omega. The 11F-like *cps* sequences have been deposited in GenBank (accession nos. MF140334 and MF140335 for PMP1342 and PMP1343 respectively).

### Quellung serotyping

Quellung serotyping was performed as described previously [10] using antisera from the Statens Serum Institut (SSI) (http://www.ssi.dk/ssidiagnostica). Briefly, pneumococcal growth from an overnight pure culture was collected from an agar plate to make a slightly turbid saline suspension. Using an inoculation loop, 1 μL was placed on a microscope slide and mixed with 1 μL of antisera. The sample was then viewed under the microscope (×400 magnification) for cells with an enlarged or swollen appearance, indicative of a positive reaction.

### Serotyping by latex agglutination

Serotyping by latex agglutination was performed with latex reagents prepared using SSI antisera and adapted from a previously described method [22]. A saline suspension of the pneumococcal culture was prepared to a density equivalent to a 4 or 5 McFarland standard. Using a glass slide, 10 μL of the bacterial suspension was mixed with 10 μL of the latex reagent containing the SSI antisera of interest. The slide was then incubated on an orbital shaker for 2 minutes at ∼140 rpm before observing the reactions for agglutination.

## Results

As part of a pneumococcal carriage survey in Fiji, we have conducted serotyping by DNA microarray on 2455 pneumococcal cultures derived from aliquots of nasopharyngeal swabs obtained between 2012 and 2015. Of these, 106 (4.3%) contained pneumococci that typed as 11F-like by microarray, which detects all 16 genes from the 11F *cps* locus (*wzg, wzh, wzd, wze, wchA, wchJ, wchK, wcyK, wcwC, wcrL, wzy, wcwT, wcwU, wzx, gct, wcjE*). This was based on similarity to serotype 11F *cps* gene content but with divergence in the *wcwC* and *wcrL* genes. 11F-like pneumococci were found in participants sampled both before and after PCV10 introduction, and across all age groups tested (19/517, 3.7%, in 5- to 8-week-olds, 28/847, 3.3%, in 12- to 23-month-olds and 52/897, 5.8%, in 2- to 7-year-olds, as well as 7/194, 3.6%, in adult caregivers). 11F-like was the most predominant member from serogroup 11, with only a small number of isolates identified as 11A by microarray (5/2455, 0.2%) and with 11B, 11C, 11D and 11F not detected.

**Fig. 1:**
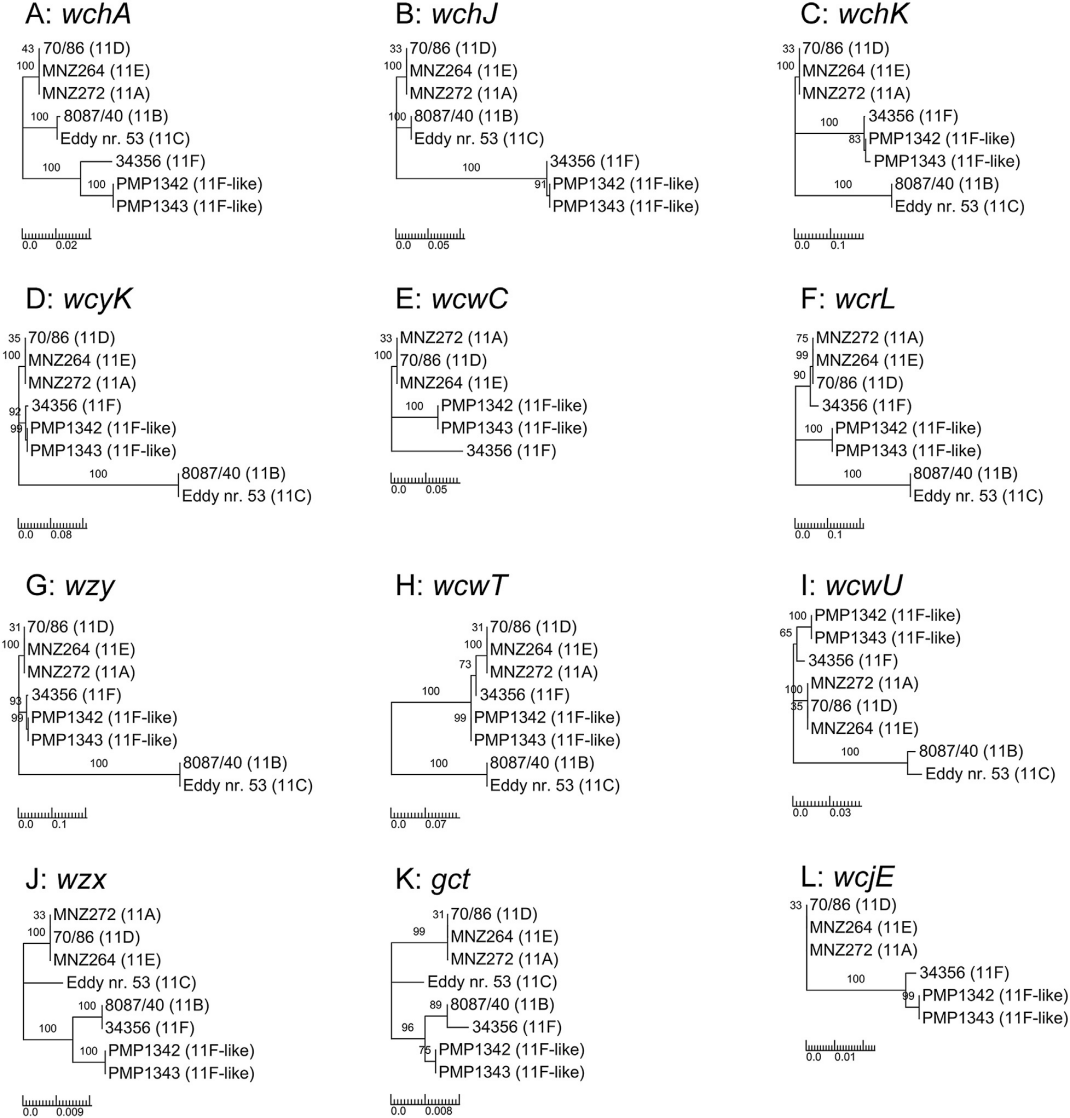
Phylogenetic analysis of serogroup 11 capsular polysaccharide genes from 11F-like isolates (PMP1342 and PMP1343) with serogroup 11 strains MNZ272 (11A), 8087/40 (11B), Eddy no. 53 (11C), 70/86 (11D), MNZ264 (11E) and 34356 (11F), (GenBank accession nos. GU074952, CR931654, CR931655, CR931656, GU074953, CR931657 respectively). Genes specific to serogroup 11 include; *wchA* (A), *wchJ* (B), *wchK* (C), *wcyK* (D), *wcwC* (E), *wcrL* (F), *wzy* (G), *wcwT* (H), *wcwU* (I), *wzx* (J), *gct* (K) and *wcjE* (L). Trees for *wcwC* and *wcjE* do not include sequences from 11B and 11C as these serotypes lack these genes. Using MEGA 6 package [21], DNA sequences were aligned using MUSCLE; and maximum likelihood of phylogeny trees were generated based on Tamura-Nei model. Scale bars represent number of substitutions per site. Statistical support for branches was determined by bootstrapping (1000 replicates) and are displayed as percentages.

To investigate and confirm the genetic basis underlying the divergence in the *S. pneumoniae* 11F-like *cps* sequences, the genomes of representative 11F-like isolates, PMP1342 and PMP1343 (Supplementary Table S1), were sequenced. Pairwise sequence alignments comparing 11F and 11F-like serogroup 11–specific genes confirmed that most genes were highly homologous (96–99% identical at the DNA level) (Supplementary Table S2). Two 11F-like *cps* genes, *wcwC* and *wcrL,* exhibited less similarity (81.8% and 84.4% respectively) (Supplementary Table S2), supporting the microarray results that indicated these genes as the source of the genetic divergence from the 11F *cps* locus. To validate these findings, phylogenetic analyses were performed comparing serogroup 11–specific *cps* genes of 11F-like isolates with *cps* gene sequences from other serotypes in serogroup 11 (11F, 11A, 11B, 11C, 11D, 11E). Only serogroup 11 sequences were used in this analysis. In the case of *wcrL,* this is because this gene is unique to serogroup 11. Although *wcwC* is present in non–serogroup 11 serotypes (7F, 7A, 22F, 22A), their level of DNA sequence identity to the 11F-like *wcwC* was significantly lower (the highest identity score to a non–serogroup 11 sequence was 60.6%, compared to over 81% with sequences from serogroup 11 serotypes). In addition, the microarray did not detect any similarity to other *cps* genes from other serotypes. The majority of serogroup 11–specific 11F-like *cps* genes were most closely related to the canonical 11F locus (Fig. 1) except *wcwC* (Fig. 1(E)) and *wcrL* (Fig. 1(F)), which did not cluster with any serogroup 11 sequences. The genetic divergence of *wcwC* and *wcrL* was also evident using a Bayesian model to infer phylogeny (Supplementary Fig. S1). This supports the serotype call made by microarray and identifies *wcwC* and *wcrL* as the divergent genes yielding the 11F-like result.

**Fig. 2:**
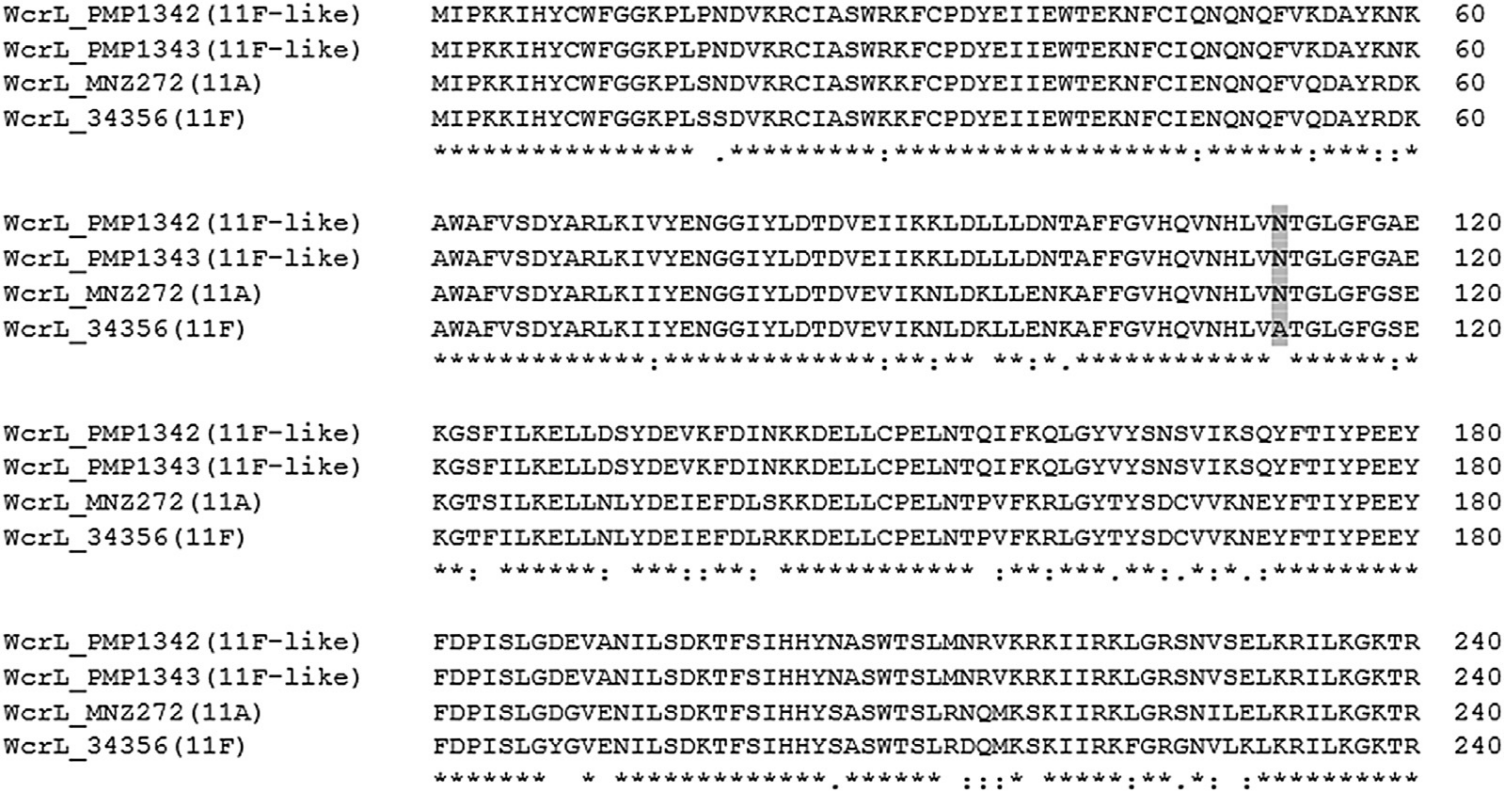
Alignment of 11F-like WcrL amino acid sequence with 11A and 11F sequences generated by Clustal Omega. Identical residues are indicated with an asterisk; conserved residents, a colon; and semiconserved residues by a period respectively. Highlighted is residue 112 (A, alanine; N, asparagine).

The *wcrL* gene encodes a glycosyltransferase involved in the transfer of the fourth sugar residue (either α-*N-*acetylglucosamine (αGlcNAc) or glucose (αGlc)) to the capsular polysaccharide repeat unit [23]. Supporting the phylogenetic analysis, *in silico* translation of 11F-like *wcrL* sequence revealed significant sequence diversity from the 11F WcrL polypeptide sequence (82% identity) (Fig. 2). It is plausible that this divergence could affect the transferase activity of WcrL and therefore the antigenic properties of the 11F-like capsule. To determine this, we performed Quellung serotyping. The 11b factor serum reacts with serogroup 11 capsules that contain αGlcNAc (11F, 11B, 11C, 11D) [24]. No Quellung reaction was observed when 11F-like isolates were mixed with the 11b factor serum (Table 1).

**Table 1:** Phenotypic serotyping for 11F-like isolates and Statens Serum Institut (SSI) reference strains (11F, 11A, 11B, 11C and 11D)

Factor serum	Major capsular antigen detected [23]	11F	11A	11B	11C	11D	PMP1342 (11F-like)	PMP1343 (11F-like)
11b	αGlcNAc	+	−	+	+	+	−	−
11c	Glycerol-1-phosphate (Gro1P)	−	+	−	+	+	+	+
11f	O-acetylation of α-Gal-H2	−	−	+	+	−	−	−
11g	Ribitol phosphate (Rib-ol)	+	−	+	−	−	−	−

Serotyping was performed using Quellung reaction as previously described [10], with ‘+’ and ‘−’ indicating a positive and negative reaction respectively with factor sera from SSI (http://www.ssi.dk/ssidiagnostica).

**Fig. 3:**
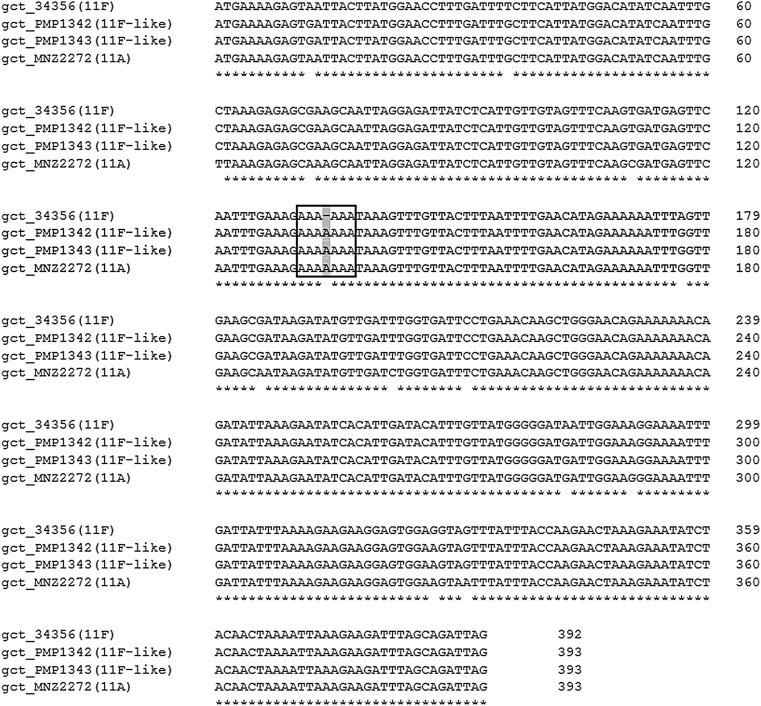
Alignment of 11F-like *gct* DNA sequences with 11A and 11F sequences generated using Clustal Omega. Identical residues are indicated with an asterisk. Box represents homopolymeric region with mutation site.

Serogroup 11 capsules have one of two phosphopolyalchohol pendants, glycerol-1-phosphate (Gro1P) or ribitol phosphate (Rib-ol). In the serogroup 11 *cps* locus, *gct* encodes a CDP-glycerol synthetase, which catalyzes the biosynthesis of Gro1P. Serotype 11F and 11B capsules lack Gro1P as a result of a single nucleotide deletion in *gct* that results in a frameshift [25]. Although phylogenetic analyses of 11F-like *gct* sequences were most closely related to the 11F and 11B *gct* sequences (Fig. 1(K)), the open reading frame of the 11F-like *gct* sequence is intact (i.e. with an additional nucleotide present) (Fig. 3). An intact *gct* open reading frame suggests Gro1P would be present in the 11F-like capsule. This predicted change in phenotype was validated by Quellung reaction using factor sera 11c and 11g, which recognize Gro1P (11A, 11C, 11D) and Rib-ol (11F, 11B) respectively [24]. The 11F-like capsule reacted with 11c but not 11g, thus confirming the presence of a Gro1P phosphopolyalchohol pendant (Table 1).

**Fig. 4:**
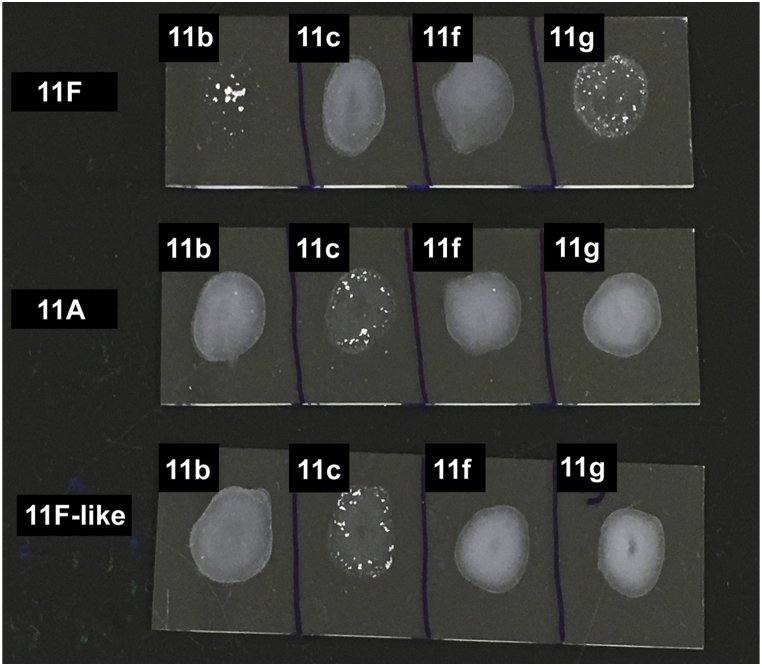
Representative latex agglutination reactions of Statens Serum Institut (SSI) 11F and 11A reference strains, and an 11F-like isolate from this study. Latex reagents were prepared using SSI antisera (11b, 11c, 11f, 11g) as Quellung as previously described [22].

Upon testing the 11F-like isolates with all serogroup 11 factor sera, it was evident that 11F-like isolates serotype as 11A by Quellung reaction (Table 1). This finding was confirmed by conducting latex agglutination serotyping (which uses the same factor sera as Quellung reaction) on six additional 11F-like isolates, all of which typed as 11A (Supplementary Table S3). Representative results of these agglutination reactions are shown in Fig. 4. Therefore, although they contain a *cps* locus most closely related to serotype 11F, minor genetic changes in this locus have resulted in the ability of 11F-like isolates to synthesize a capsular polysaccharide resembling that of serotype 11A based on cross-reaction with typing antisera.

## Discussion

In this study, we identified and characterized a new pneumococcal serotype variant from a vaccine impact study in Fiji. Genotypic serotyping of these isolates by microarray identified a *cps* locus that is closely related to the 11F *cps* locus but genetically divergent as a result of sequence variation within the *wcwC* and *wcrL* genes. Quellung reaction and latex serotyping revealed that the 11F-like capsule phenotypically serotypes as 11A (Fig. 4 and Table 1).

11F-like capsules lack αGlcNAc and contain Gro1P, demonstrating that the genetic differences between 11F and 11F-like *cps* loci yield capsules with differing antigenic properties.

The substrate affinity and glycosyltransferase activity of WcrL varies among serotypes within serogroup 11 and is determined by the amino acid at position 112. WcrL enzymes with alanine at this position transfer αGlcNAc (11F, 11B, 11C), those with asparagine transfer αGlc (11A), and those with serine transfer both (11D) [23]. In contrast to the 11F WcrL, the 11F-like WcrL possesses an asparagine at this position (Fig. 2). This explains why no Quellung or latex agglutination reaction occurred with the 11b factor sera (which detects αGlcNAc) (Table 1 and Fig. 4), as the 11F-like WcrL would not be able to transfer αGlcNAc, resulting in the 11F-like capsule lacking this modification.

The CDP-glycerol synthetase encoded by *gct* catalyzes the synthesis of Gro1P, which is subsequently incorporated into the capsule repeat unit by WcwU. In serotypes 11B and 11F, *gct* does not encode a functional enzyme as a result of a single nucleotide deletion, explaining the absence of Gro1P in their capsules. While this has been reported previously [25], the site of the deletion has not been described; nor has the mechanism by which it may have occurred been postulated. We identified that the deletion is located within a homopolymeric region of seven tandemly repeated adenosine nucleotides (Fig. 3). Sequences such as this can be unstable and subject to slipped-strand mispairing during DNA replication, resulting in the insertion or deletion of a single nucleotide. Such spontaneous frameshift mutations have been reported previously, including within homopolymeric adenosine repeat regions of pneumococcal virulence genes *pspA* and *spxB* [26]. In serogroup 15, slipped-strand mispairing of a TA dinucleotide repeat in the *wciZ* gene is the basis for the structural differences between 15B and 15C capsules [27]. Thus, we propose that the single nucleotide deletion in the *gct* gene of 11F and 11B is the result of a slipped-strand mispairing event and explains the difference in the phosphopolyalchohol pendant of the capsule of these serotypes with 11A, 11C and 11D.

In the case of the 11F-like *gct* gene, our phylogenetic analysis identified it is closely related to the 11F and 11B *gct* genes (Fig. 1(K)). In comparison to the 11F and 11B sequences, the 11F-like *gct* gene contains a single nucleotide insertion in the homopolymeric region (Fig. 3). We hypothesize that this insertion occurred by slipped-strand mispairing and restored the open reading frame, allowing Gro1P synthesis and its subsequent incorporation into the capsule, explaining why these isolates react with 11c factor sera (which detects Gro1P) (Table 1 and Fig. 4). Alternatively, it is possible that 11F-like is a closely related progenitor of 11F in which the slipped-strand mispairing event has not yet occurred. Interestingly, *gct* homologues are present in the *cps* locus of serogroups 16 and 18, and serotype 45, which also possess similar homopolymeric regions and may therefore also be subject to slipped-strand mispairing.

Our genetic analyses also identified the acetyltransferase gene *wcwC* as a source of divergence in the 11F-like sequence (Fig. 1(E) and Supplementary Fig. S2). WcwC is responsible for O-acetylation of galactose in the capsular polysaccharide subunit [24]. Interestingly, genetic variants that emerge within other serotypes that have phenotypic consequences have been attributed to nonsense mutations in acetyltransferase genes [12], [13], [14], [27]. However, the 11F-like *wcwC* gene still contains an intact open reading frame. It remains to be elucidated whether divergence in the *wcwC* sequence translates to changes in acetylation patterns in the 11F-like capsule.

The microarray platform uses a high level of stringency for the detection of *cps* genes because a high level of identity (above 90%) is required to conclude that a particular *cps* gene from a specific serotype is present. In the case of the isolates described in this study, they were designated with the ‘like’ annotation because the divergence in *wcwC* and *wcrL* (Fig. 1(E) and (F)) meant that they did not meet the sequence identity criteria to be called 11F. Microarray can discriminate between serotypes 11A and 11F because of the *wchJ* and *wchK* genes, which are highly divergent between these serotypes. The 11F-like *wchJ* and *wchK* genes were more similar to the 11F homologues compared to 11A (Fig. 1(B) and (C) and Supplementary Table S2), which is why the microarray called the variants identified in our study 11F-like and not 11A-like.

As we progress through the genomics era, the benefits provided by genetic approaches such as microarray to infer pneumococcal serotype are making these methods an attractive alternative to the more laborious phenotypic methods. However, as demonstrated in our study, it is important to note that this will not always be reflective of serotype, especially with the existence of genetic variants that are yet to be discovered. Given that multiple genetic approaches to pneumococcal serotyping are commonly utilized, it is important to consider how such methods (in addition to microarray and DNA sequencing) would report the 11F-like variant, which genetically resembles 11F across most of the *cps* locus. The US Centers for Disease Control and Prevention recommend a qPCR-based approach using primers and a probe for 11A/11D that target the *wzy* gene of the *cps* locus [28]. When we performed this qPCR, both the 11A SSI reference strain and our 11F-like isolates were detected (Supplementary Table S4). In addition, these primers and probe also detected the 11F SSI reference strain (Supplementary Table S4), suggesting this qPCR method cannot distinguish between 11A, 11D, 11F and 11F-like. To our knowledge, this qPCR system has not previously been validated against serotype 11F. The 11F and 11F-like *wzy* sequences contain very few mismatches in the annealing sites of these primers and probe (Supplementary Fig. S3). Therefore, it is not surprising that this qPCR would also detect serotype 11F.

Another qPCR method, described by Sakai *et al.* [29], uses primers and probe targeting the 11F *wchK* gene, which does not detect 11A. This 11F-specific qPCR also detected the 11F-like isolates (consistent with the observation that the 11F-like *wchK* gene was most closely related to the 11F sequence, Fig. 1(C)), leading to a mistyping of these variants (Supplementary Table S4). Kapatai *et al.* [30] described a bioinformatic tool (PneumoCaT) that can be used to infer serotype from whole genome sequence data. In the case of serogroup 11, the pipeline is designed such that it can detect the small genetic differences between serotypes such as *gct* allele (in frame or frameshifted) and the codon starting at position 334 in *wcrL* (that specifies the amino acid at position 112). As a result, when the sequence reads of the 11F-like isolates were run through the PneumoCaT pipeline, they were correctly typed as 11A (score 5/5; presence of *wcwC* and *wcjE,* absence of *wcwR,* intact *gct* ORF and *wcrL* codon starting at position 334 as AAU, which specifies N at amino acid position 112). Overall, it is evident that most genetic approaches to pneumococcal serotyping would mistype the variants described in our study as 11F unless they take into account relevant small variations. Therefore, although genetic approaches to serotyping are advantageous, it is important that they do not completely replace phenotypic methods. Serologic methods provide insights into the antigenic properties of capsules produced by different serotypes—important knowledge to be taken into consideration for vaccine formulation.

Our study highlights the use of microarray for identification of genetic variants of *S. pneumoniae* in clinical samples. The data from this study will be used to update microarray slides to accurately identify 11F-like variants in future studies to accurately measure prevalence. Of interest, we have identified nasopharyngeal isolates from Lao PDR and Mongolia that also typed as 11F-like by microarray, suggesting that this variant may be widespread. We have identified that serotyping calls for serogroup 11 from molecular approaches can differ from those using phenotypic methods. This has implications for pneumococcal disease surveillance and monitoring serotype replacement after vaccine introduction. Ultimately, a combination of genotypic and phenotypic pneumococcal serotyping methods may be needed at reference centers to detect pneumococcal serotype variants and fully characterize the effect of vaccination on pneumococcal epidemiology.
