# Supplementary material for: A novel genetic variant of Streptococcus pneumoniae serotype 11A discovered in Fiji
Source: Clin Microbiol Infect. 2018 Apr;24(4):428.e1–7. doi: 10.1016/j.cmi.2017.06.031 (PMC5869949; doi:10.1016/j.cmi.2017.06.031)
Supplement: Supplementary file 1 [file mmc1.docx]

**Supplementary data**

**Supplementary Table S1.** 11F-like isolates used in this study.

| Isolate | Origin | Optochin sensitivity | Serotype (microarray) | Multi-locus sequence type |
| --- | --- | --- | --- | --- |
| PMP1342 | Nasopharynx of a 12-23 month old Fijian child | Sensitive | 11F-like | 99 |
| PMP1343 | Nasopharynx of a 12-23 month old Fijian child | Sensitive | 11F-like | 99 |

**Supplementary Table S2.** DNA sequence identity scores from pairwise alignments of 11F-like serogroup 11-specific cps genes from other serotypes.

|  | Identity score (%) | | | | | |
| --- | --- | --- | --- | --- | --- | --- |
| Gene | **11F** | **11A** | **11B** | **11C** | **11D** | **11E** |
| *wchA* | 96.3 | 93.6 | 93 | 92.9 | 93.6 | 93.6 |
| *wchJ* | 99.6 | 78.9 | 78.7 | 78.7 | 78.9 | 78.9 |
| *wchK* | 99.2 | 82.3 | 65.2 | 65.2 | 82.3 | 82.3 |
| *wcyK* | 98.9 | 96.4 | 65.7 | 65.7 | 96.4 | 96.4 |
| *wcwC* | 81.8 | 86.5 | - | - | 86.5 | 86.5 |
| *wcrL* | 84.4 | 85.5 | 66.7 | 66.7 | 85.3 | 85.5 |
| *wzy* | 98.9 | 95.7 | 64.3 | 64.3 | 95.7 | 95.7 |
| *wcwT* | 98.8 | 96.8 | 72 | 72 | 96.8 | 96.8 |
| *wcwU* | 98.1 | 97.5 | 89.6 | 89 | 97.5 | 97.5 |
| *wzx* | 98.4 | 97.2 | 98.4 | 96.9 | 97.2 | 97.2 |
| *gct* | 98.5 | 97.7 | 99 | 98.2 | 97.7 | 97.7 |
| *wcjE* | 99 | 96.9 | - | - | 96.9 | 78.1 |

**
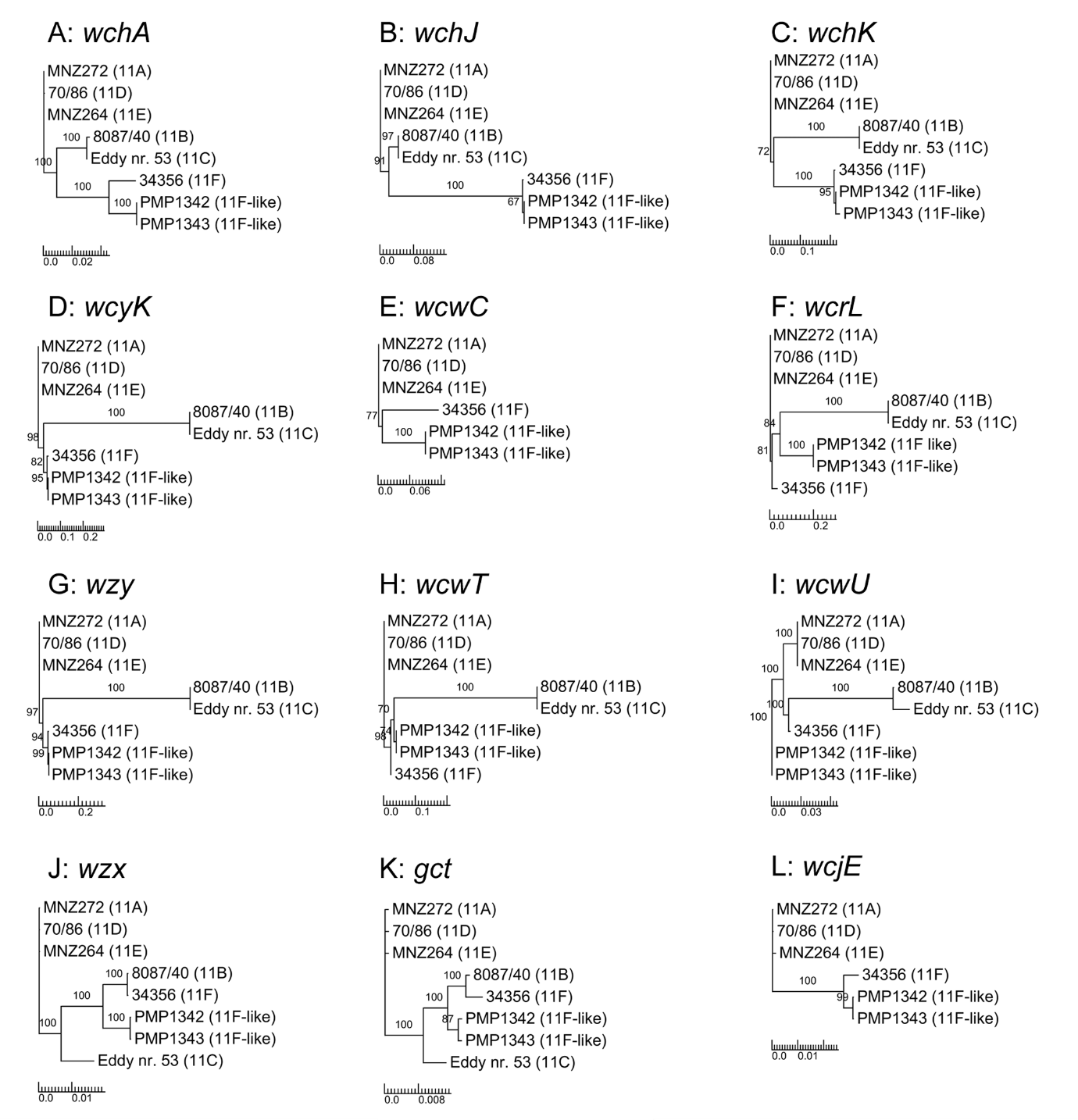
**

**Supplementary Figure S1.** Bayesian analysis of phylogeny of serogroup 11-specific *cps* genes from 11F-like isolates with serogroup 11 isolates. Serogroup 11-specific genes include; *wchA* (A), *wchJ* (B), *wchK* (C), *wcyK* (D), *wcwC* (E), *wcrL* (F), *wzy* (G), *wcwT* (H), *wcwU* (I), *wzx* (J), *gct* (K) and *wcjE* (L). DNA sequences were aligned using MUSCLE and trees were generated using MrBayes 3.2.3 for 2x10^4^-3.2x10^6^ generations for a standard deviation of split frequencies of <0.01.

**Supplementary Table S3.** Serotyping of 11F-like isolates by latex agglutination (using reagents prepared from SSI antisera as previously described [1]).

|  | Factor serum | | | |
| --- | --- | --- | --- | --- |
| Isolate | **11b** | **11c** | **11f** | **11g** |
| PMP1337 | **-** | **+** | **-** | **-** |
| PMP1338 | **-** | **+** | **-** | **-** |
| PMP1339 | **-** | **+** | **-** | **-** |
| PMP1340 | **-** | **+** | **-** | **-** |
| PMP1341 | **-** | **+** | **-** | **-** |
| PMP1344 | **-** | **+** | **-** | **-** |

WcwC_PMP1342(11F-like) --MLQNLKLIKRRLKPIKRKLKPIKRTYKKIVYSITKSNMRKRLKNTDFSIISDNCWGGR 58

WcwC_PMP1343(11F-like) --MLQNLKLIKRRLKPIKRKLKPIKRTYKKIVYSITKSNMRKRLKNTDFSIISDNCWGGR 58

WcwC_MNZ272(11A) MKGKSFLLKMLQNLKPIKRKLKPIKRTYKRIVYSITKSNMRKRLKNTDFSIISDNCWGGR 60

WcwC_34356(11F) ----------MKTLKSIKRKLKPIKKTYKKIVYSITKSKMRKRLKNRDFSIISDNCWAGR 50

: ** *********:***:********:******* **********.**

WcwC_PMP1342(11F-like) VYEELGLPYRTPFIGLYIFSEDYVKLLKNFKKYMEYELTFTNNSKWNTEYDGEYPIGILN 118

WcwC_PMP1343(11F-like) VYEELGLPYRTPFIGLYIFSEDYVKLLKNFKKYMEYELTFTNNSKWNTEYDGEYPIGILN 118

WcwC_MNZ272(11A) VYEELGLPYRTPFIGLYIFSEDYVKLLKNFRKYMEYELTFTNNSKWNTEYDGEYPIGILK 120

WcwC_34356(11F) VYEELGLPYRTPFIGMYIYSEDYVKLLKNFKDYMGYELTFTNVTKWNEEYTGEYPIGILK 110

***************:**:***********:.** ******* :*** ** ********:

WcwC_PMP1342(11F-like) DIEIHFLHYSSMEEAYKKWNKRKKRINYKNIFFKMNDDNKCSLKLLKEFDTLDLKNKIIF 178

WcwC_PMP1343(11F-like) DIEIHFLHYSSMEEAYKKWNKRKKRINYKNIFFKMNDDNKCSLKLLKEFDTLDLKNKIIF 178

WcwC_MNZ272(11A) DIELHFLHYANQEEAYEKWNKRKNRINYENIFFKMNDDNKCSLKLLKEFDTLDLKNKIIF 180

WcwC_34356(11F) DIEIHFLHYVNQEEAYEKWNKRKNRINYENIFFKMNDDNKCSFELLKEFDNLDFKNKIIF 170

***:***** . ****:******:****:*************::******.**:******

WcwC_PMP1342(11F-like) AAFNYKELDNLIHFTNPIGNGNVGADLFYYNKYFDVVNWLNGEY-- 222

WcwC_PMP1343(11F-like) AAFNYKELDNLIHFTNPIGNGNVGADLFYYNKYFDVVNWLNGEY-- 222

WcwC_MNZ272(11A) AAFNYKELDNLIHFTDPRGNGNVGTDLFYYDQYFDVVNWLNGETQT 226

WcwC_34356(11F) AAFNYKELDNLIHFTNPRGNGNVGTDLFYYDQYFDVVNWLNGETQT 216

***************:* ******:*****::***********

**Supplementary Figure S2.** Clustal omega alignment of WcwC amino acid sequences from 11F-like, 11A and 11F serotypes. Residues denoted as ‘*’ indicate identical residues, while conserved and semi-conserved residues are specified by ‘:’ and ‘.’, respectively.

**Supplementary Table S4.** qPCR detection of pneumococcal serogroup 11 using primers and probes designed by Pimenta et al. [2] and Sakai et al. [3]. qPCRs were performed using the Brilliant III Ultra-Fast qPCR master mix (Agilent technologies) as per the manufacturer’s instructions using the probe and primer concentrations specified previously [2,3] and ~1 ng/µl of genomic DNA added as the template. Reactions were subjected to initial denaturation at 95°C for 3 mins, followed by 40 cycles of 95°C for 15 sec and 60°C for 30 sec. qPCRs were performed on two independent occasions with representative Ct values from one experiment displayed.

|  |  | Mean cycle threshold (Ct) value | |
| --- | --- | --- | --- |
|  |  | ***wzy 11A/11D [2]*** | ***wchK 11F [3]*** |
| Strain | **Serotype** |  |  |
| SSI 11F | **11F** | **24.43** | **25.23** |
| SSI 11A | **11A** | **22.88** | **No Ct** |
| SSI 11B | **11B** | **No Ct** | **No Ct** |
| SSI 11C | **11C** | **No Ct** | **No Ct** |
| SSI 11D | **11D** | **24.14** | **No Ct** |
| PMP1342 | **11F-like** | **23.68** | **24.56** |
| PMP1343 | **11F-like** | **23.96** | **24.96** |
| No template control | **n/a** | **No Ct** | **No Ct** |

A: 11A/11D *wzy* forward primer

11A/11D-F ---------------------------------------------------------AAA

wzy_34356(11F) ACTTTTAATGGTCGATACCAAATTTGGCGCATTGTGTATGCTACCATTCCTCAAGTGAAA

wzy_MNZ272(11A) ACTTTTAATGGTCGATACCAAATTTGGCGCATTGTGTATGCTACCATTCTTCAAGTGAAA

wzy_PMP1342(11F-like) ACTTTTAATGGTCGATACCAAATTTGGCGCATTGTGTATGCCACCATTCTTCAAGTGAAA

***

11A/11D-F TGGTTTGGATATGGTTTGTTTGG-------------------------------------

wzy_34356(11F) TGGTTTGGATATGGTGTGTTTGGCTTTCAATTTAAACTTCCGTGGCAGGAATTGGGAGAA

wzy_MNZ272(11A) TGGTTTGGATATGGTTTGTTTGGCTTTCAATTTAAACTTCCGTGGCAGAAATTGGGAGAA

wzy_PMP1342(11F-like) TGGTTTGGATATGGTGTGTTTGGCTTTCAATTTAAACTTCCGTGGCAGGAATTGGGAGAA

*************** *******

B: 11A/11D *wzy* reverse primer

11A/11D-R -------------------------------------------------AGTGCTAACTG

wzy_34356(11F) ATAAAAAATCATAATAAGGAATGAGATAATCCCAACAATTCCACTATCAAGTGCTAACTG

wzy_MNZ272(11A) ATAAAAAATCATAGTAAAGAATGAGATAATCCCAACAATACCACTATCAAGTGCTAACTG

wzy_PMP1342(11F-like) ATAAAAAATCATAGTAAAGAATGAGATAATCCCAACAATGCCACTATCAAGTGCTAACTG

***********

11A/11D-R TAAAACTTGATTATGAG-------------------------------------------

wzy_34356(11F) TAAAACTTGATTATGAGTATAATTTATTCCAATTTCTCCCAATTCCTGCCACGGAAGTTT

wzy_MNZ272(11A) TAAAACTTGATTATGAGTGTAATTTATTCCAACTTCTCCCAATTTCTGCCACGGAAGTTT

wzy_PMP1342(11F-like) TAAAATTTGATTATGAGTATAATTTATTCCAATTTCTCCCAATTCCTGCCACGGAAGTTT

***** ***********

C: 11A/11D *wzy* probe

11A/11D-Probe -------------------------ATTCCAACTTCTCCCAATTTCTGCCACGG------

wzy_34356(11F) TAAAACTTGATTATGAGTATAATTTATTCCAATTTCTCCCAATTCCTGCCACGGAAGTTT

wzy_MNZ272(11A) TAAAACTTGATTATGAGTGTAATTTATTCCAACTTCTCCCAATTTCTGCCACGGAAGTTT

wzy_PMP1342(11F-like) TAAAATTTGATTATGAGTATAATTTATTCCAATTTCTCCCAATTCCTGCCACGGAAGTTT

******* *********** *********

D: 11F *wchK* forward primer

11F-F -----------------------------------TGGTCCAGCTACTTTTATGGC----

wchK_34356(11F) TTTATTGAGGAATCTGATACAATTGTTACCCATGGTGGTCCAGCTACTTTTATGGCAATT

wchK_MNZ272(11A) TATATAGATGAAGCAAATATTATCATTACACATGGCGGCCCAGCTACATTTATGGCAGTT

wchK_PMP1342(11F-like) TTTATTGAGGAAGCTGATACAATTGTTACCCATGGTGGTCCAGCAACTTTTATGGCAATT

** ***** ** ********

E: 11F *wchK* reverse primer

11F-R ----------------------------------------TGATCATTCACATGCTCCCC

wchK_34356(11F) ATACCCTTCAGAGGATATTTTAGAACAAAAATTCATCTGATGATCATTCACATGCTCCCC

wchK_MNZ272(11A) ATACCCTTCGGATAGTATTTTAGAACAAAATTCCATCTGATGATCATTCACATGCTCACC

wchK_PMP1342(11F-like) ATACCCTTCAGAGGATATTTTAGAACAAAAATTCATCTGATGATCATTCACATGCTCCCC

***************** **

F: 11F *wchK* probe

11F-Probe ---------------ACTCCAATAGTTGTTCCGAGGCAAAAGA-----------------

wchK_34356(11F) ATTGCTAAAGGAAAAACTCCAATAGTTGTTCCGAGGCAAAAGAAATTTGGGGAGCATGTG

wchK_MNZ272(11A) ATTGCTAAAGGAAAAATACCAATAGTTGTTCCGAGATTAAAGAAGTTTGGTGAGCATGTG

wchK_PMP1342(11F-like) ATTGCTAAAGGAAAAACTCCAATAGTTGTTCCGAGGCAAAAGAAATTTGGGGAGCATGTG

* ***************** *****

**Supplementary Figure S3**. Comparison of annealing sites of primers and probes used for qPCR to detect serotypes 11A/11D and 11F from Pimenta et al [2] and Sakai et al [3], respectively. Mismatches to the primer or probe are highlighted.

References:

1. Porter BD, Ortika BD, Satzke C. Capsular serotyping of *Streptococcus pneumoniae* by latex agglutination. JoVE 2014; 91:e51747.
2. Pimenta FC, Roundtree A, Soysal A, Bakir M, du Plessis M, Wolter N, et al*.* Sequential triplex real-time PCR assay for detecting 21 pneumococcal capsular serotypes that account for a high global disease burden. J Clin Microbiol 2013; 51:647-52
3. Sakai F, Chochua S, Satzke C, Dunne E.M, Mulholland K, Klugman K.P, et al. Single-plex quantitative assays for the detection and quantification of most pneumococcal serotypes. PLoS One 2015; 10:e0121064.
